# Editorial: Genetic and molecular markers in human viral infections

**DOI:** 10.3389/fcimb.2026.1854930

**Published:** 2026-05-12

**Authors:** Maria Alice Freitas Queiroz, Jordana Grazziela Coelho-dos-Reis, Antonio Carlos Rosário Vallinoto

**Affiliations:** 1 Laboratory of Virology, Institute of Biological Sciences, Federal University of Pará, Belém, Brazil; 2 Laboratory of Basic and Applied Virology, Federal University of Minas Gerais, Belo Horizonte, Brazil

**Keywords:** biomarkers, genetics, human viral infections, immunity, virulence

Although significant progress has been made in controlling viral infections over the past few decades, diseases caused by viruses still pose a major public health challenge worldwide (Holmes et al., 2024). Furthermore, the development of severe and debilitating chronic conditions, such as those caused by HIV-1, HTLV-1, HBV, HCV, and SARS-CoV-2 have not been fully understood. Many viruses have mutations that help them evade the host immune response, aiding their persistence (Beachboard and Horner, 2016). Immune response dysregulation can result in an ineffective antiviral attack or an excessive inflammatory response, leading to acute illnesses with high mortality or viral persistence, which is responsible for chronic diseases (Christiaansen et al., 2015).

Genetic variations in both viral and host genomes can trigger alterations in the immune response and determine either favorable or unfavorable prognoses for viral infections. These genetic alterations as a result of high mutation rates may lead to immune evasion. Genetic alterations in key molecules of the host response can influence the intensity of the antiviral and inflammatory responses and, ultimately, impact viral susceptibility and symptomatic manifestation. The response to infections differs among individuals, resulting in infections of varying intensities, which may lead to asymptomatic, severe and fatal cases.

The topic “*Genetic and molecular markers in human viral infections*” provides an overview of genetic alterations associated with modifications in molecules and mechanisms of viral activity and/or the human host’s immune response. These markers can be utilized to identify pathogenic viral agents, improve the understanding of the virus-host response, and determine disease prognosis and treatment efficacy. In this topic, the study by Longo et al. described the first documented case of progressive multifocal leukoencephalopathy (PML) associated with BKPyV in a patient with erythrodermic mycosis fungoides treated with mogamulizumab, a monoclonal antibody that significantly alters the immune response. The association between BKPyV and PML was established through brain magnetic resonance imaging, which revealed multifocal white matter lesions compatible with PML, and the detection of BKPyV DNA in plasma, urine, and cerebrospinal fluid (CSF). Sequencing of the viral protein 1 (VP1) and non-coding control region (NCCR) of BKPyV showed compartment-specific VP1 sequence variability and NCCR structural rearrangements in CSF isolates. These findings highlight the intra-host heterogeneity of BKV, which may contribute to viral adaptation and neurotropic potential, emphasizing the need for vigilant monitoring of both BKPyV and JCPyV during mogamulizumab treatment (Longo et al.).

The study conducted by Zhou et al. investigated the detection levels of hepatitis B virus RNA (HBV RNA) in serum and its clinical significance in patients with chronic hepatitis B (HBV) before and after treatment. Analysis of patient samples showed a higher HBV RNA positivity rate compared to viral DNA and HBeAg. Untreated patients exhibited higher HBV RNA and DNA, whereas in the treatment group, the HBV RNA rate was higher. Treated CHB patients presented significantly lower serum levels of HBV RNA, DNA, HBeAg, HBsAg, ALT, and AST compared to the untreated group. In untreated CHB patients, elevated HBV RNA levels were correlated with higher levels of ALT, AST, HBsAg, HBeAg, and HBV DNA. The authors suggested that HBV RNA exhibits greater sensitivity and precision, potentially serving as a useful tool for monitoring treatment efficacy and assessing viral persistence (Zhou et al.).

Regarding HTLV infection, Ferreira et al. investigated, for the first time, the association of *TLR3* gene polymorphisms (rs5743305 T/A and rs3775291 C/T) with HTLV-1 infection concerning susceptibility, manifestations of infection-associated diseases, receptor expression levels, proviral load, and inflammatory and antiviral cytokines. Furthermore, the authors observed no association between the *TLR3* rs5743305T/A polymorphism and HTLV-1 infection, the presence of symptoms, or TLR3 and IFN-α levels. However, regarding the *TLR3* rs3775291 C/T polymorphism, asymptomatic individuals carrying the TT polymorphic genotype presented significantly higher IFN-α levels and lower proviral loads. The profile of asymptomatic individuals with polymorphic genotypes for *TLR3* rs3775291C/T was characterized by higher levels of TLR3 and IFN-α and lower levels of proviral load, TNF-α, and IL-6 compared to those with the wild-type genotype. All in all, this indicates that the *TLR3* rs3775291C/T polymorphism appears to contribute to a better clinical progression of HTLV-1 infection and the inflammatory process in asymptomatic individuals (Ferreira et al.).

Zhu et al. conducted the first study to explore comprehensively the impact of IFN-λ polymorphisms on Influenza A virus (H1N1 variant) infection. The authors also highlighted the importance of IL-29 and IL-28 cytokines in combating viral infection and noted that the age factor may affect both viral dissemination and therapeutic responses. In experiments using human alveolar epithelial cells (HAEs) from young donors, they observed higher viral replication and a poor response to IL-29 antiviral treatment. Single nucleotide polymorphisms (SNPs) in the IFNL genes have been associated with H1N1 infection outcomes. HAEs from donors with rs12979860TT and rs8099917GG genotypes showed higher replication rates and did not respond to antiviral therapy with IL-29. Cells with the rs12979860TT genotype also produced lower levels of IFN and showed inhibited IL-29 promoter activity in response to H1N1 infection. The study underscored that the IFN-λ signaling axis is a potential therapeutic target and revealed that polymorphisms in the *IFNL* gene may influence H1N1 infection characteristics, providing a basis to guide the development of more effective anti-influenza treatments (Zhu et al.).

Li et al. evaluated influenza in children using a non-targeted metabolomic approach to investigate small molecule metabolites in peripheral blood that were associated with the disease. By comparing these metabolites with those of healthy children, potential biomarkers for early detection and diagnosis of influenza were explored. In the group of children infected with the H1N1 variant, alterations in 14 glycerophospholipid metabolites were identified compared to the control group; of these, 11 metabolites showed a marked reduction and were correlated with lower levels of inflammatory markers, including WBC, neutrophils, C-reactive protein and procalcitonin. In the group of children infected with the H3N2 variant, 12 glycerophospholipid metabolites were significantly altered, 9 of which showed reduced expression. Two primary metabolites stood out: LysoPC(20:0/0:0), which was downregulated and positively correlated with ALT but negatively correlated with WBC count; and LysoPA(18:1(9Z)/0:0), which was upregulated and correlated with increased levels of ALT, AST, and LDH. The authors demonstrate that H1N1 and H3N2 infections are characterized by distinct metabolic profiles in children and that specific glycerophospholipid metabolites are associated with inflammatory and liver function markers, highlighting them as potential biomarkers for disease monitoring and early diagnosis (Li et al.).

Regarding SARS-CoV-2 infection, Abudouleh et al. investigated the correlation between SARS-CoV-2 mutations found in blood samples and various alterations in the fibrinolysis system, as well as disease severity based on clinical outcomes and the need for ICU admission. The authors observed the presence of SARS-CoV-2 RNA in 27.6% of the blood samples. The N501Y, D614G, K417N, and P681R mutations, located in the SARS-CoV-2 spike protein, were associated with higher ICU admission rates. Omicron (BA.1.1) variant genes were associated with thrombosis in unvaccinated hospitalized COVID-19 patients with comorbidities. Plasma levels of tPA, aPTT, and D-dimer were significantly higher in participants carrying the N501Y mutation. Thrombosis was the most prevalent condition among patients with severe COVID-19; therefore, elevated levels of TAFI, PAI-1, and tPA associated with the disease may have potential as prognostic markers for hypofibrinolysis (Abudouleh et al.).

The manuscripts published in this edition stand out for their diversity of information, focusing on viral gene variations that can positively influence host adaptation or persistence. Moreover, the articles are within the scope of this topic and enrich the literature regarding the dysregulation of immune molecules involved in disease progression, and genetic polymorphisms in components that determine the immune and inflammatory responses associated with infection susceptibility and disease development. Since viruses are under constant selective pressure, genetic mutations have emerged and were more frequently identified in recent years. These mutations potentially modify the host response to infection and disease pathogenesis. Therefore, the investigation of molecular and genetic alterations in human viral infections must be continuous, vis-à-vis an effective molecular surveillance of emerging and reemerging virus that pose public health threat worldwide.
